# Brain's geometries for movements and beauty judgments. A contribution of topos geometries

**DOI:** 10.3389/fpsyg.2025.1583185

**Published:** 2025-09-04

**Authors:** Daniel Bennequin, Alain Berthoz

**Affiliations:** ^1^Université Sorbonne Paris Cité, Paris, France; ^2^Collège-de-France, Paris, France

**Keywords:** beauty, brain, geometry, movement, perception, topos, stack, multiplicity of spaces

## Abstract

We present a theory on the neural basis of aesthetic experience, and judgment of beauty. It is based on both empirical facts concerning brain mechanisms and theoretical mathematical theories. We first recall previous evidence that the brain uses several non-Euclidian geometries for perception and action at different scales of space (personal, peri-personal, near locomotor, environmental, imaginative). This is supported by neuroscience data (brain imaging, neuropsychology, movement control, etc.). For example, the movement of drawing obeys specific kinematic rules, that reflect the control by Euclidian and affine geometries. We already formulated the corresponding geometries in brain's networks by using Topos and Stacks theory of the mathematician Alexander Grothendieck. The present article extends the previous proposals by suggesting that a meta-geometry provides the binding between these specialized geometries, by using known higher structures and dynamics (like n-Topos and n-Stacks) for joint perceptions and movements, and other modalities, as concepts, memories or emotions, at different spatial scales domains. We suggest that a form, an object, a movement, an environment, an event, an idea, is perceived as beautiful if the data provided by the senses and programs are embedded in these higher geometries, providing a sort of dynamic recognition, through relations of generalized proportions.

## Introduction

### A brief historical perspective

A traditional view on the perception of beauty came from Plato, who insisted upon the role of configurations of symmetries and color. Aristotle identified measure and order as being crucial for experience of beauty, reformulating the theory of Plato of generalized symmetry. The word symmetry in ancient Grece had the meaning of the many relations between the parts of an entity. The theory presented here insists upon two major ideas. The first idea is that a form or object, or idea, which is qualified, or experienced, as aesthetic or beautiful, is always perceived as having movement or motion and related to a repertoire of Euclidian and non-Euclidian movement related geometries. The role of movement was mentioned in 1616 by François de Sales (1969) “*But as for animate and living things their beauty is not accomplished without good grace, which, besides the propriety of perfect parts, which makes beauty (of the fixed); adds the propriety of movements, gestures and actions, which is like the soul and life of the beauty of living things…*.” Movement influences deeply the judgment of beauty and reciprocally the emotional valence of a stimulus does influence temporal goal-oriented movements (Esteves et al., 2016).

The second idea is that the judgment, or experience of beauty (or its contrary ugliness) is not reserved to visual (or auditory, or tactile) impressions, it can be also attributed to entities (beings) that have no dimensions, nor visual representation, like some events, behaviors, decisions, ideas or theories. This is compatible with the view of the Greek philosopher Plotin (or Plotinus) who, against the Gnostics, rightly observed that for him, the judgment of beauty can rely on sensations and perceptions, not necessarily visual, and relies on the harmony between different ideas, or between ideas and what they represent, e.g. words related to things or events. For Plotin, the judgment of Beauty relies on the elevation to Reason. Plotin wrote a treatise about “beauty of intelligible.” He also insisted that higher level of beauty correspond to an adequation between the inner world and the external one (cf. Plotin, 2021). This is similar to suggestion made in our own theory: elevation to a higher order of dynamical structures as proposed by Plotin.

A very clear mention, of the importance of motion and some higher dynamical form of geometry is present in *The analysis of beauty* by the painter Hogarth (2010). His *magic curve*, “his line of beauty,” evokes liveliness and movement. For him beauty comes from the evocation of motion, and is related to life. Six principles are necessary conditions of perception of beauty: fitness, variety, regularity, simplicity, intricacy, quantity. The forms are full, they are not diagrams or real lines, they are movements themselves, rotations and translations, imagined or felt.

As a last example in a vast litterature, Diderot (1751) in his Encyclopedia article “Beau,” extended the view of Plato and Aristotle about symmetries and proportions. He quotes St Augustine idea that beauty is an *immediate perception of ratios*, not only of measurable proportions. Beautiful is everything that evokes the idea of ratios in the intellect (entendement). What we suggest is very similar to what Diderot suggested. We consider the role of geometries, their variety, and idendify different levels of beauty. We also propose that “beauty” refers to *higher sets or categories*, forming generalized geometries for action spaces, cf. Appendix 1. We shall relate our geometrical theory of beauty with goal-oriented movement and action.

We will suggest that the feeling of beauty, or the judgment of “beauty,” is attributed to something, an object, an idea, when this thing evokes an *internal geometry* of the brain (in the sense we will describe below). It implies an homology, in the sense of *relation between relations* (not necessarily an isomorphism) between an inner state (or event) of mind and a state (or event) of the perceived, or imagined world. We also will suggest that a multiplicity of inner spaces and geometries on them is necessary and must be conceived dynamically, but the compatibility of the different processes must be maintained. It's a problem similar to that posed for vision, where objects are broken down into attributes: color, movement, place etc., but perceived as a whole. It is still debated whether the unified perception of an object's identity is due to a “grandfather neuron,” or to synchronizations in the coding of the different attributes. More simply, we can assume that at each level of composition of elements of information, something new is added in the process, like a predisposed reservoir of forms for assembling the elements, supported by specific dynamics of neuronal assemblies.

We have proposed elsewhere (Bennequin and Berthoz, 2017) that for the preparation and execution of movements, new kinds of brains geometries are necessary, without points, made by topos in the sense of Grothendieck, developed in XXth century for the needs of arithmetic and algebraic geometry (cf. Appendix 1). Our model was that in the brain, the information flows in geometrized spaces over a family of categories, with enlargement of geometry when information sources are joined. The geometries of topos (or more generally of fields of n-stacks), allow us to describe operations, at several levels (essentially three), guiding the *adaptation* of movements and their combinations (here, rapid neural adaptation to a context is mainly concerned.).

We hypothesize now that the feeling of beauty in front of a scene, corresponds to the access of our brain's operations to several “levels” of geometries, able to join movement, emotion, memory and other aspects of inner life.

We cannot here review the vast amount of work done on the behavioral or neuronal basis of the experience and judgement of beauty. But we have selected a few results relevant to our own approach.

### Behavioral evidence

Most of the available studies deal with visual perception and aesthetic cognitive and emotional experience of beauty (Stamkou et al., 2024). Gustav Fechner (1856) claimed that beauty results from immediate pleasure, see (Brielmann et al., 2021). Recent evidence (Brielmann and Pelli, 2019) has shown that indeed, intense beauty requires intense pleasure. In addition, follow-up repeated measures showed that shared taste contributed 19% to beauty-rating variance, only one third as much as personal taste (58%). Addressing age-old questions, these results indicate that feeling beauty is a kind of pleasure, and that beauty is more personal than universal.

However, pleasure is not a sufficient characteristic of beauty, as shown by the attribution of “good” or “testy,” and not beautiful, for odors and tastes. In order to respect the meaning of beauty in different cultures, it is necessary to characterize better the associated feelings reports. Experimental results were lacking until recently. First convincing results are described by Brielmann et al. (2021). Six dimensions are statistically significant: (1) Intense pleasure; (2) impression of universality; (3) wish to continue the experience; (4) exceeding expectation; (5) perceived harmony in variety; (6) meaningfulness. The authors underline that this agrees with the views of Plato and Kant about universality (contrary to Hume). Surprise, considered by Aristotle and Hegel, is discarded; it is even detrimental to beauty, (Santatyana, 1896), see (Brielmann et al., 2023). Note that harmony and variety belong to the aesthetic list of Hogarth, but not the other aspects.

It was shown that curved and angular shape of visual forms were rated pleasing and harmonious if the stimuli consisted of a few lines that were clearly discernible (Stanischewski et al., 2020). This was in accord with neurophysiological data suggesting that different brain networks treated, respectively, shape and texture (upcit).

An important question concerns the difference between attention and perception of beauty. Children seem to attribute more attention to symmetrical patterns, relative to similar but asymmetrical patterns, but they showed no explicit preference for those patterns (Huang et al., 2018). The aesthetic experience of biological beauty is dictated by inherited brain concepts, which are resistant to change even in spite of extensive experience. The experience of artifactual beauty on the other hand is determined by post-natally acquired concepts, which are modifiable throughout life by exposure to different experiences. The experience of mathematical beauty (Ishizu and Zeki, 2011) is consistent with one of the characteristics of the biological categories, namely a lesser variability (Zeki et al., 2018). Note a paradoxical bias toward positive judgement (particularly of beautiful) of works with negative content (Menninghaus et al., 2017).

### Neurophysiological mechanisms

Several atemps have been made to establish a brain based theory of the experience of beauty and in particular Semir Zeki has provided several studies on this approach.

#### Contribution of the visual system

Elementary constituents of neuronal receptive fields of the primary visual areas in mammals are based on positions, directions, textures (preferential excitation by grids movements), and colors. The first result of visual integration is to put them in a whole, making composition, adding coherence, creating types (cf. area IT for the first forms, and V8 for colored figures). This is typical of the first level of neural mechanisms for attribution of beauty to a visualy perceived form. For instance, there seems to be evidence that curvature is involved. A region in early visual cortex (BA 17) encompassing largely areas V2-V3 is sensitive to variation in computational curvature across both beauty judgments and approach-avoidance decisions, whereas a region encompassing the fusiform gyrus (BA 37) exhibits sensitivity to perceived curvature only when participants made beauty judgments (Vartanian et al., 2024).

Some studies deal with the attribution of beauty to human face, for example, greater aesthetic judgment was attributed to the mobile and communicative parts for the female face, but by contrast, to the rigid, structural, parts for the male face (Øvervoll et al., 2020). Brain imaging results suggest that better memory for attractive faces reflects greater interaction between a region associated with reward, the orbitofrontal cortex, and a region associated with successful memory encoding, the hippocampus (Tsukiuraa and Cabeza, 2011). While the beauty of faces convergently activated the left ventral striatum, the beauty of visual art convergently activated the anterior medial prefrontal cortex (aMPFC). This activation of the prefrontal cortex was confirmed in another study (Cela-Conde et al., 2004). For a relation with the default mode network, see Cela-Conde et al. (2013). And for the relation with the mu-rythm, see Umilta et al. (2012).

However, a conjunction analysis failed to reveal any common brain regions for the beauty of visual art and faces (Chuan-Peng et al., 2020).

#### Movement and judgment and experience of beauty

As stated in the introduction, our theory suggests that movement is essential in the experience of beauty. Experimental evidence has been provided by a number of studies. For example, compared with no action observation, aesthetic judgments of calligraphy images with action observation elicited stronger activation in the anterior cingulate cortex and the bilateral insula. Meanwhile, the superior parietal lobe which is associated with relevant inner action imitation, was also activated when observing the creator's action. Brain activation in the superior parietal lobe, anterior cingulate cortex, and the bilateral insula indicated that observing the creative action of the creator contributed to the aesthetic experience of the observer (He et al., 2021).

In aesthetic judgments, the medial and lateral subdivisions of the orbitofrontal cortex as well as subcortical stations associated with affective motor planning (globus pallidus, putamen–claustrum, amygdala, and cerebellar vermis), are engaged, whereas the motor, premotor and supplementary motor areas, as well as the anterior insula and the dorsolateral prefrontal cortex, were engaged by both aesthetic and affective judgments (Ishizu and Zeki, 2013). The hedonic state associated with activation of right dorsal anterior insula underpines aesthetic experience for art work. It therefore seems that specific brain “motor” or at least dynamic networks, not usually involved in movement control, are involved in the experience of beauty.

More generally, dynamic scenes induce more activation in aesthetic experience than static scenes in several related regions of the brain (Zhao et al., 2020). In this study, static and dynamic landcapes where shown, the occipital lobe, frontal lobe, supplementary motor area, cingulate cortex and insula were commonly activated both in the aesthetic judgments of dynamic and static landscapes. But stronger activations of middle temporal gyrus (MT/V5), and hippocampus were found in the aesthetic judgments of dynamic landscape.

### Computational models

Some studies concern the preference for certain sensory patterns by eveluating the quatity of information level and the influence of temporal rythms and symmetry. Models based upon Fisher information (i.e. the structure of the lower bounds of variance in estimation) have been proposed (Grzywacz and Aleem, 2022). Computational algorithms have also ben used to identify image properties thought to have a role in aesthetic appeal for visual stimuli (Brachmann and Redies, 2017). Others attempt to distinguish between aesthetic and basic non aesthetic sensory processing: in one model (Rezaul Karim et al., 2022), an *aesthetics-only* channel primarily involves *restricted local processing* for quality or richness (e.g., attractiveness, beauty/prettiness, elegance, sublimeness, catchiness, hedonic value) analysis, whereas a *perception-to-aesthetics* channel involves *global/extended local processing* for basic feature analysis, followed by a *restricted local processing* for quality or richness analysis. In another study (Pombo and Pelli, 2023), it was shown that the perception of beauty is not different of normal non aesthetic sensory motor perception, from the point of view of Information theory (the Shannon statistical approach). However, the theory of *fluency* suggests that beauty is determined by the efficiency of information processing in the perceiver's brain (Yoo et al., 2023) (Here fluency is the likelihood to reproduce the stimulus from the inner state in a neural network.). This theory was supported by Dibot et al. (2023), using experiments with faces and a sparse artificial neural network (sparse means low density of connectivity). The fluency theory, joined to learning theory, is used in the computational model of Brielmann and Dayan (2022). A great advantage of this model is to permit to take into account interindividual differences and changes in time (Brielmann et al., 2023). It is obvious that universal models are not adequate and sufficient. One must consider interindividual differences. Therefore, the above model deserves to be praised. We will come back to this important question at the end of the present article.

## Internal brain geometries

None of the above approaches has really been able to propose a theory on the neural foundations of perception and experience of beauty.

Our hypothesis is that the qualification of “beautiful” expresses that the perceived object or form, or event, or thought, belongs to a geometry that is linked to our cerebral processes for processing internal spaces. These spaces and their geometries (specific transformations acting on a space (cf. Appendix 1, and Bennequin and Berthoz, 2017), are mathematical models for the brain processes to prepare, initiate and control body movements, but they also underlie a transposition of these processes into other sensory-motor domains (e.g., color, form, actions, …). Examples are Euclidian classical or log-polar coordinates (Tabareau et al., 2007), The internal geometries in these spaces are linked to transformations in the external world, and related transformations of the inner world, which correspond generally to virtual rather than possibly real transformations of the external world. The judgment of beauty expresses the feeling that the concerned stimulus belongs to such a geometry, or to generalized ones combining them, as we will describe later. This is a way for extending the notion of using perception of ratios, mentioned by the tradition, from Plato and Aristotle to Diderot. But it corresponds also to the idea of Plotin, that in beauty something in the world meets deeply ourself.

The first part of the following section reviews the main known examples of such geometries, involving networks of brain areas, generally multimodal. In appendices (cf. Supplementary document) we describe the mathematical models that we had used: transformations, groups, categories, functors, then topos and stacks (networks of coherent geometries). We also introduce 2-categories (for describing networks of networks), and 3-categories, corresponding to “higher-order geometries,” that allow to decribe the influences of modalities one over the others (emotion, memory, cognition, learning).

### Geometries for adaptation

An important function of the neuronal networks in a brain's area, helped by other systems of cells, glia or neuromodulators, is to shape the inner information flow (sensori-motor and inside world). When the incoming messages, or external or internal conditions, are changed, the area has to preserve or optimize this function. One important manner to achieve that consists to transform the cells activities correspondingly. These transformations are made by using information and action at different scales, of spatial extent and time delays, from microns and milliseconds to centimeters and hours. The complexity of these processes is such, that evolution and development use a repertory of chosen standard transformations, depending on individual cells, but involving drifts and shifts between cells, in a cooperative manner. At this point, it is advantageous to use a geometry.

A clear example is the space of color: in the thalamus and the primary visual areas, a virtual space is created and used to adapt the color preferences of sensitive cells, in particular to a change of ambient light. It has the form of a 3D affine space (cf. Derrington et al., 1984). Affine transformations (linear plus translation, cf. Appendix 1) compensate for changes in the world through changes in the activities of neural ensembles (cf. Webster and Mollon, 1995). In the same manner that voluntary movements compensate for spatial displacements, according to Poincaré (1902). In deeper areas, the color field is complicated by (semi-)local effects analogs to a curvature of surfaces in differential geometry (Coxeter, 1969).

### The brain's multiple (functional) geometries for controlling voluntary movements

The idea that action is at the foundation of geometry and of the concept of space is not new. Poincaré (1902) wrote that to localize a point in space is simply to imagine the movement necessary to reach it. It is not a question of representing the movements themselves but simply the muscular sensations which accompagny them. He also insisted upon the idea that the notion of space arises from the fact that the identity of objects stems from our movements relatively to them. There is no *a priori* space, but exploitation of a set of transformations, described by groups (cf. Appendix 1).

We find the elements of Euclidian geometry in many regions of the brain: motor areas like M1, Basal Ganglia, Cerebellum, vestibular sensors and central nuclei, and at the “end” of the visuo-vestibular flow of information, in the parahippocampal region, where are situated Place cells, Head direction cells and Grid cells, but also the cells for boundaries, corners, velocities, durations (Rowland et al., 2016; Moser et al., 2017) that are elements for Dynamics and Topology (as developed by Poincaré himself in his fundational works, on Dynamical systems and Analysis Situs). This system is known to serve navigation in ambient space.

However, several other geometries contribute to sensation, perception and action in space. A departure of perceived space from being merely Euclidian was suggested also by the pioneering works of Jan Koenderink (Koenderink and van Doorn, 1991; Todd et al., 2001; Koenderink et al., 2002), Luneburg and Heelan cf. (Heelan, 1983) also suggested that optical space is not totally different from a geometrized (homogeneous) space, although it is in no way close to being Euclidian. They also considered models where the curvature changes from being elliptic in near space to hyperbolic in far space (then non-homogeneous). Rudrauf et al. (2020) explained several optical illusions (Moon illusion, Ames room, …) by changes of 3D projective frames in the brain.

Euler (1707–1783) (cf. Coxeter op.cit.) understood that a coherent affine geometry can be defined outside the Euclidian geometry, and is well-adapted to study several kinds of ratios without notion of length. During the hisory of mathematics and their applications, the scope of geometry was considerably enlarged. Starting with Euclidean geometry, based on rigid displacements and mirror symmetries, the elements of affine geometry, forgetting about size and angle, but retaining the notion of parallelism of straight lines, were recognized in Arabic sciences and developed by Descartes (XVII th century). Then a full affine geometry was explicitely formulated by Euler in eighteen century. Poncelet et al. (XIX-th century) invented Projective geometry, further extending the allowed transformations to optical effects, like anamorphosis (Baltrusaitis, 1969), by including points at infinity (introduced by Kepler, and Desargues) in a space, larger than the affine space. Affine geometry contributes to organize the generation of voluntary movements, writing and locomotion, in particular the relation between shape and timing. We did not know where in the brain can be detected affine areas: higher visual areas like OT, premotor areas, cerebellum? nucleus NST?

For movement and perception, it has been shown that the brain uses multiple (functional) geometries for controlling voluntary movements. These are essentially guides for the adaptation of these movements. Several internal geometries intervene together for the movement of an effector in the same space, 2D or 3D physical space (Bennequin et al., 2009), through variation of geometries and their compositions. For example the law which states that linear velocity is proportional to the radius of curvature to the power of 1/3 (named 2/3 law because of the resulting angular velocity), was experimentally discovered by Lacquaniti et al. (1983). Its validity, stability and possible origins are discussed by Zago et al. (2018); in particular it is proved here that it is not a statistical artifact. The 2/3 law is pertinent for hand movements and for locomotion (Vieilledent et al., 2001). It can be interpreted as the kinematical law invariant by the “equi-affine” geometry, that which respects area as well as being affine, according to Hanzel and Flash (1999) and Pollick and Sapiro (1997) (It means that if a curve is transformed in equi-affine manner, the transported law conserves the same form). The group of this geometry (Appendix 1), the so-called “special affine” group, is the only one of co-dimension 1 in the affine group. Based on this special geometry, we suggest that we prefer to generate piecewise parabolic trajectories because the subgroup of the affine group that respects the parabola is of dimension 2. For a straight line, it's of dimension 3, even better when possible. For the circle or a conic, it is of dimension one.

As a result, we can see that what counts is not necessarily the shortest path, or the one that saves the most energy, but the one that offers the most freedom, in order to adapt its on-line dynamics to as many conditions as possible. In fact, geometric invariance is compatible with optimization (Flash et al., 2016) combining minimum jerk (principle of maximum smoothness; Flash and Hogan, 1985; Viviani and Flash, 1995) and geometrically invariant path laws, pure or mixed. Then during the same movement, depending on the shape of the trajectory, the particular local conditions, we (and other animals no doubt) call upon several geometries. Where the trajectory is almost straight, Euclidean geometry dominates; where we need to turn quickly, equi-affine geometry prevails; and for transitions (as inflection points), pure affine geometry comes into play. This explains isochrony, the fact that two similar trajectories tend to be covered in the same time (at least if they are close in time). A number of brain imaging studies have identified the brain structures involved in these kinematic laws (Levit-Binnun et al., 2006; Casile et al., 2010; Dayan et al., 2007), for perception, seeing trajectories.

### A variety of internal spaces

#### Multiplicity of action spaces

Space is not uniquely processed in the brain but, throughout Evolution, distinct neural modules have been organized for action, perception, memory, emotion, etc. Our hypothesis is that different geometries are implemented in these various networks that need to be compatible to save the unity of mental processes. This modular organization is compatible with the principles of simplexity (Berthoz, 2012) and vicariance (Berthoz, 2016) and the basic evolution of the brain connectome and networking (Changeux et al., 2021).

#### Spatial action spaces

Well-know evidence from neurology tells us that there are several action spaces. Brain imaging studies also have shown different networks for so-called “far” and “near” action spaces. According to a vast neuropsychological literature and recent brain imaging data we have proposed to distinguish at least four mains action spaces (Berthoz, 2020).

(1) *Body space (personal space)*. This is the space of our body movements coded in a number of reference frames. A synthetic “body schema” has ben identified in the temporo-parietal junction (Carter and Huettel, 2013).
(2) *Reaching and grasping space (peripersonnal or “near action” space)*. At a fine grain a mosaic of areas: F2, F7 are involved for object location and coordination of body and arm, F5 for the hand (and mouth). Reaching, grasping, holding, manipulations: M1, F2, F3, for movements parameters, amplitude, timing, motor command … F4-VIP, for transforming object location into appropriate movements toward them, AIP for size, shape, orientation, identity, weight, visual and physical information (Michaels et al., 2020).
(3) *Near Locomotor space (extra-personal space)*. It involves a core of areas in M1, PP, BG, Thalamus, MLR (PNf, PPR), RF, Cerebellum, BSN, chord (cf. Wei et al., 2020), networks vary for different contexts (Berg et al., 2023; Pernía-Andrade et al., 2021).
(4) *Environmental and navigational space*. A number of areas have been identified for navigation and environnemental spatial memory (cf. Bermudez-Contreras et al., 2017) among which: anterior subiculum, ventral striatum, thalamus, retrosplenial cortex, parietal cortex, hippocampus, medial entorhinal cortex and neocerebellum (see also Ghaem et al., 1997).

We will detail in what follows (Appendix 2) how these spaces support new sort of geometries, without points, described by convenient *toposes* and *stacks* (cf. Appendix 1), that are deep inventions of Alexander Grothendieck, motivated by arithmetic and algebraic geometry, but which are also related to Logics.

It is also known that there is a lateralisation between the left and the right brain. The left brain is involved not only in language but also in sequential, egocentric, memory for navigation, preference to process details (and spatial high frequencies), and the right brain involved in more global, allocentric, processing of spatial information. The neural syndrome of neglect in which right parietal damage induces an ignorance of the left space although visual perception is maintained is one of the most spectacular illustrations of this lateralisation. The ability for perspective change is also lateralised as well as processing of boundaries etc. Finally lateralisation has also been shown in the cerebellum for navigation (Igloi et al., 2010; Iglói et al., 2015) and also in its contribution to disctinct networks for exploration and for exploitation in a spatial navigation path. This suggest that there is a variety of geometrical mechanisms implemented in the corresponding neural networks.

#### More abstract internal spaces

Buszaki and Moser (2017), after the discovery of grid cells in the Entorhinal cortex, conjectured that analog cells and structures exist also in the parahippocampal region for cognition, in the form of a sort of navigation in a space of concepts, feelings and thoughts. In the Hippocampus proper, cells transposing place cells, are supposed to support reasoning and probabilistic inference. Prehension space uses cells in pre-motor areas, close to mirror cells, that code for points in space, for reaching them, independently if this is made by the hand (pointing) or the gaze (saccade). To generate real movements, the neural system has to make some logical or semantic operations, that show his aptitude of abstraction (Neromyliotis and Moschovakis, 2017). Note that Georgopoulos attributed the origin of reasoning to the necessary computations in Premotor areas for generating motions in good order (Georgopoulos, 2000). We see on these examples that the geometries (here topological and Euclidian for navigation) of motion spaces, generate indirectly similar geometries for thinking.

### Models of topos and stacks. Categories, n-categories and topodynamics

#### Summary of general properties of topos and stacks (“champs” in French mathematical terminology)

In the Appendix 1, we have summarized some “modern” mathematical concepts, mathematical basis of our model of “higher geometries” in the brain. Definitions, of groups, transformations, spaces, categories can also be found in Bennequin and Berthoz (2017), and in a neighboring form in Habas et al. (2020) and Torkhani-Langlois et al. (2024). The paradox is that the new geometries appearing here, are at the same time, more complex from the mathematical point of view, but more concrete and closer to the intuition in their applications to the brain, because in them, fields replace points, dynamical connections replace figures, local preferences (and indifferences) replace fictive global representations. The reader can go to Appendix 1 for finding a discussion of Group, Category, Grothendieck topology, sieve, site, presheaf, sheaf, Topos, Geometrical space, geometrical topos, stack, champ, 2-category, functor, 3-category.

Intuitively, a *category* is made by a collection of *objects* and paths between them, named *morphisms*, or arrows, that can be composed. For instance, an oriented graph generates a category. Group is the particular case with only one object and all arrows invertibles (i.e., revertibles, as for displacements in our ambient space). A *topology* is a data of *sets* of arrows going to the objects, representing refinements of the objects. A *presheaf* is a collection of ensembles (or sets) labeled by the objects, equipped with maps lifting the arrows in the reverse directions (it is also named a contravariant functor). A presheaf is a *sheaf* when the refinements determine these sets at the goal from the sets at the sources. Both the collections of pre-sheaves and sheaves over a *site* (a category with a topology, cf. Appendix 1) have natural structures of categories, whose morphisms are coherent natural transformations, objects by objects. A *topos* of Grothendieck is any category of sheaves over a given site (see for instance Bell, 2008; Caramello, 2017).

A fundamental theorem of Giraud and Grothendieck, see (Grothendick and Verdier, 1972) asserts that all the usual constructions of Set theory (the doday basis of most of mathematics), i.e., make coherently products of families of sets, make quotients by equivalence relations, make sets of subsets and sets of mappings, are possible in any topos. Thus, a topos is a kind of complete world for natural constructions. On another side, sheaves and toposes are the tools for describing possible relations between local and global, for instance in differential, analytic or algebraic geometry. *Stacks* add the local actions of other categories, like groups, considered as internal structures. Then they give a notion of geometrical sheaves, richer than ordinary ones.

#### Dynamical levels and brain networks

The brain is a whole that works in part locally, dividing its work in networks of inner areas, but preserving the coherence of the local processes to ensure the success of its actions, in function of the goals. That is why it is so important to adapt the local and global processes in order to optimize the information flow and the execution of movements. Therefore, it is not surprizing that toposes would be welcome for describing brains dedicated networks, and that for adaptation, we need geometries on these toposes, i.e., stacks.

In the following subsection, we present how topos geometries, represented by stacks, give models for the different action spaces. In addition, the different action spaces must be coordinated. This can be obtained by building another kind of space which will combine the several actions spaces and make them compatible. We propose that in the brain it happens at least at three levels of such generalized geometries.

#### The topos level, or equivalently level 1

The simplest example of a geometric topos we have in mind is in motor control for example the arm/hand movement groupoid, which covers all rigid (i.e., Euclidian) movements in physical space, when manipulating objects. Here, for topos terminology the *site* consists of two elements (i.e., objects): a (label for configuration of arm and hand geometry), and b (label for what happens in Euclidian space), and three arrows (two self returning arrows called identities 1_a, 1_b, and a unique arrow from b to a). The internal structures for the stack (in fibers over the objects) are given, respectively, by non-holonomic transformations of the arm and the hand over a and by rigid dispalcements over b.

**Figure 1:**
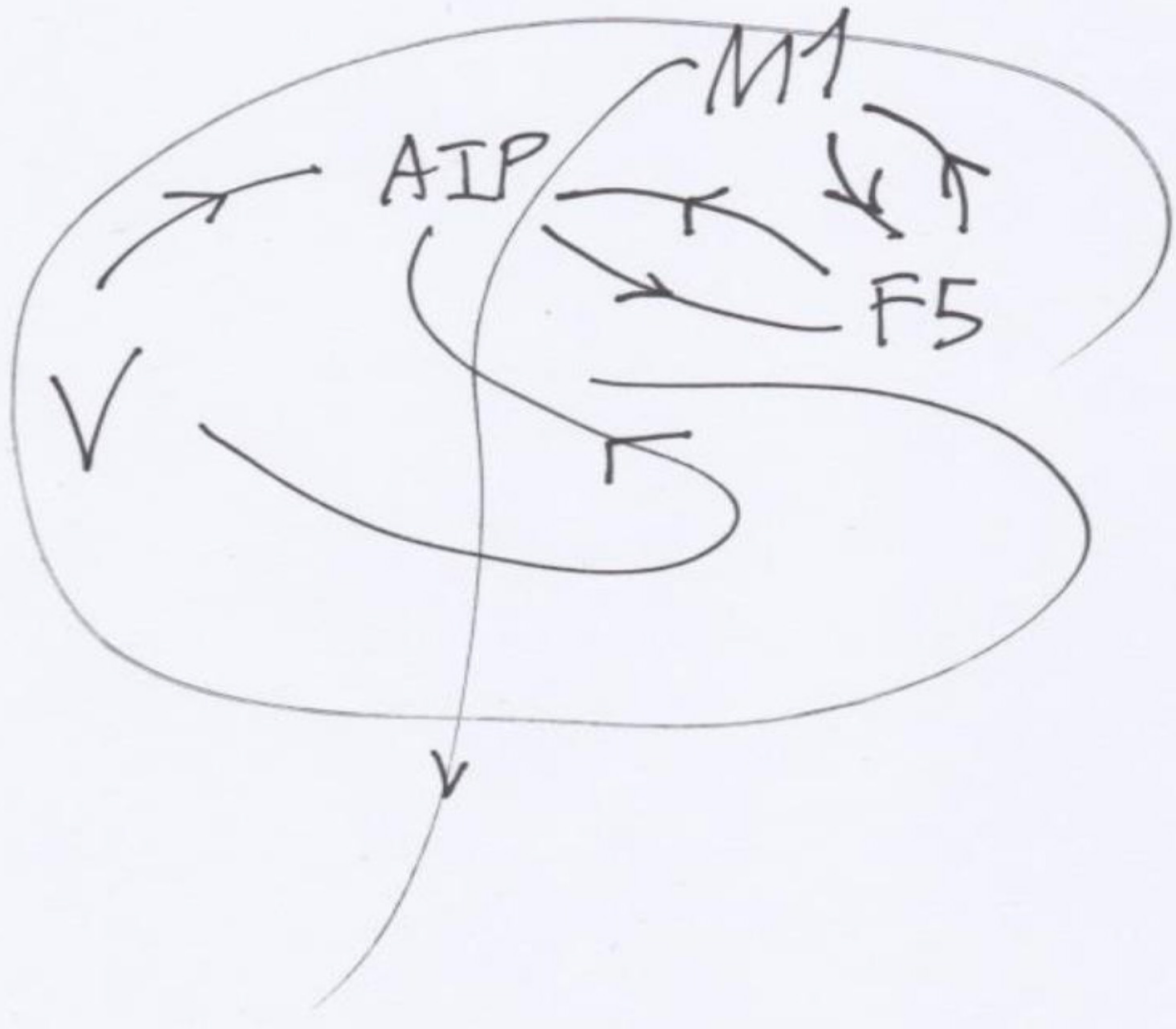
Simplified schema of the connections between brain's areas of monkeys underlying reaching and grasping. The occipital visual areas V send information to the anterior intra-parietal area AIP, reciprocally related with the rostro-ventral premotor cortex in F5, itself reciprocally related to the primary motor cortex M1. Then from M1, the descending tract goes to the brain stem and the motor neurons.

**Figure 2:**
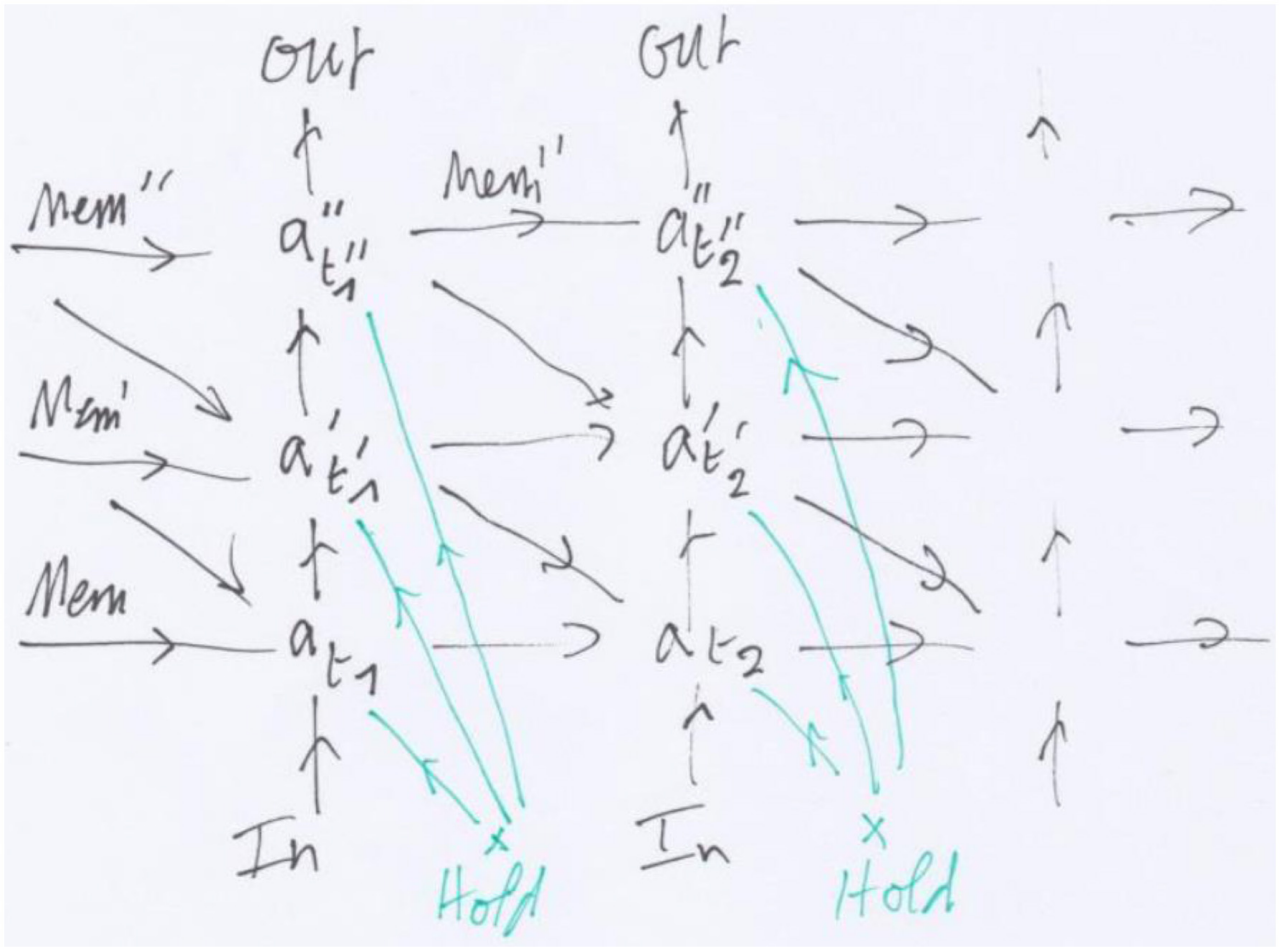
The (modular) recurrent neural network (RNN) unfolding Figure 1: horizontal arrows represent memory, vertical arrows the feedforward propagation and horizontal arrows the feedback. The transversal arrows in green represent a Hold signal before movement.

In order to take into account, the properties of the 3D objects in the world it is necessary to take into account more than one or two areas, say three elements a, a', a” linked together by neural connections, each possessing a specific group (or a category) of transformations of its activities G, G', G”, so that their respective actions are compatible, and adaptable (cf. Figure 1). So, the activities of all three areas together are to be taken into account, at different instants of time. The good way to realize (artificially) such a dynamic, is a “recurrent neural network” or RNN (cf. Appendix 2) (cf. Figure 2).

**Figure 3:**
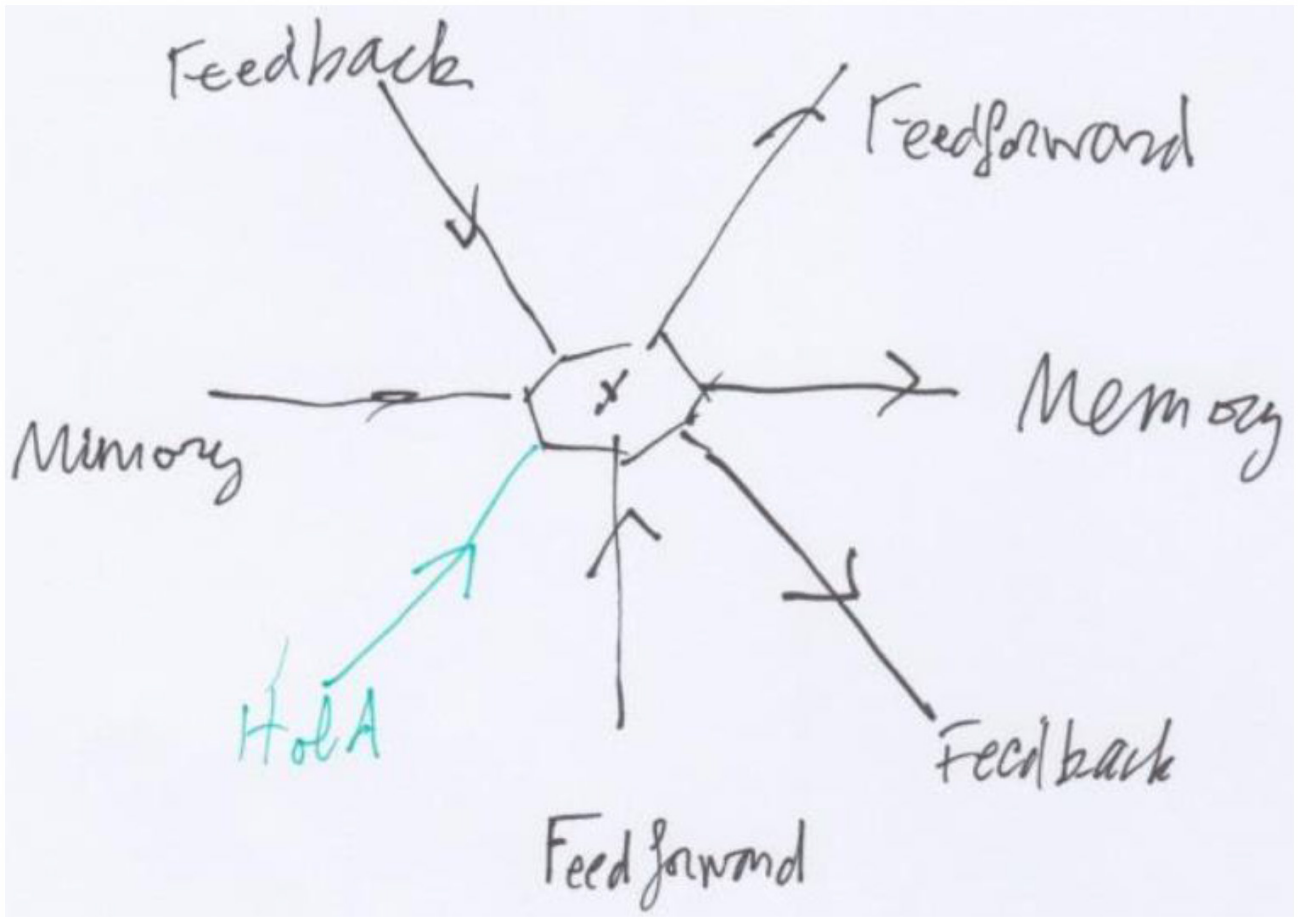
The generic vertex of the RNN network of Figure 2, with four incoming arrows and three outgoing arrows.

**Figure 4:**
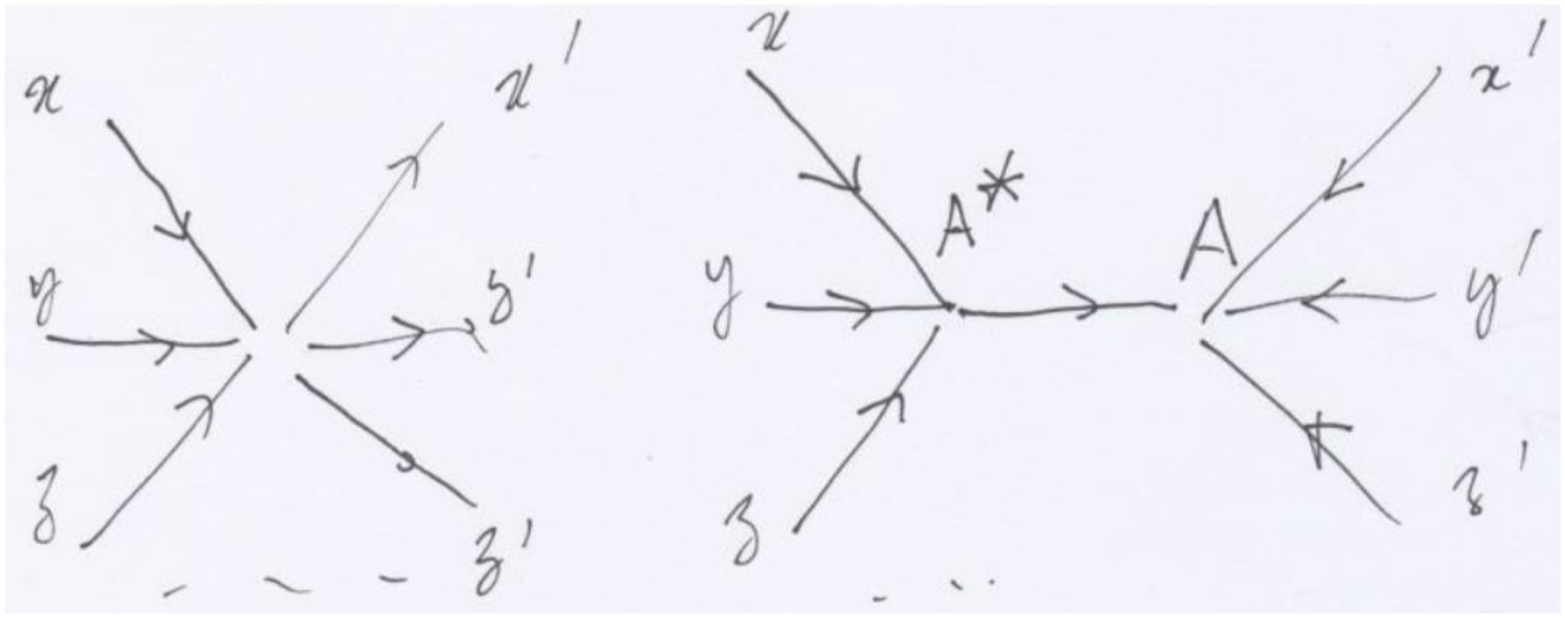
The local surgery transforming a vertex into a fork, where the vertex is replaced by two vertices and one arrow. Over this last arrow we impose an isomorphism in the sheaf. The topology at A* contains the covering by incoming arrows, implying that the value of the sheaf in A* is the product of its values in x, y, z.

For example, we can consider the arm, the hand, and a goal to grasp, aided by the visual system. Here the inner categories G', G” describe, respectively, hand and finger posture and arm posture, and G describes the properties of the objects. All these contribute together to the known variety of frames (body-centered, hand-centered, eye-centered, object-centered, and so on) that govern the action pattern (cf. Figure 3). In order to construct an associated topos, we shall use a particular case of what is developed in the first chapter of Belfiore and Bennequin (2022) and the neurophysiological exposition of the grasping network in monkeys by Michaels et al. (2020) (see Appendix 2) (cf. Figure 4).

**Figure 5:**
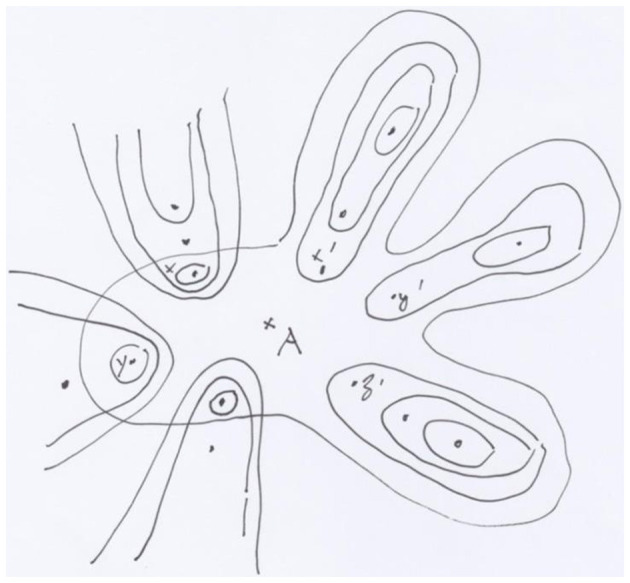
A finite site with ordinary topology defining the same topos as the surgeries. The close contours represent the open sets.

Having constructed the adequate topos, the flow of activities is an object X of this topos T. It is also proved in Belfiore and Bennequin (2022) that the repertory of dynamics for the network (called its weights), also defines an object in T, and that the learning gradient flows is a morphism in T. The advantage of creating such a topos is that all the operations on the neural network or between several such networks, become *natural*, from both the logical and geometrical points of view. This naturalness is a condition in order to be used by the brain. (For instance, we can consider the addition of new neurons.). Another advantage was to introduce natural places in the network for inner decisions (cf. Figure 5).

At this level 1, it is possible to create analog toposes for the other mentioned action spaces, Body, Locomotion, Navigation.

A construction of (toposic) level 1 can also represent what happens for the mixtures of geometries in drawing or walking (Bennequin et al., 2009): the populations of several areas are divided in subregions A,A', A”, …, controled by the different groups of plane geometry (affine, equiaffine, Euclidean), but after M1 and before the muscular connection, in the descendant path to the chord, they jointly project to the same assembly of neurons, say B, giving a signal of velocity that combine the purely geometrical velocities.

#### The 2-category level, or level 2 of geometry

To describe more complex actions, such as grabbing an object with the hand while walking around a room, we need to take into account several topos and functors of one into the other. For that we will use 2 and 3-categories (cf. MacLane, 2010).

The fundamental example of 2-category in Mathematics, on which our intuition must be based to make this structure intelligible, is that of the 2-category of topological spaces, with continuous applications as arrows (or 1-cells) and homotopy classes (i.e., continuous deformations) of homotopies between two applications as 2-cells. Then morphisms of morphisms are homotopies. In general, a 2-category is made by a collection of objects, and for any pair of objects a, b, a category Mor(a,b) of 2-morphisms from a to b, whose objects are named horizontal arrows, and morphisms between two arrows are named vertical arrows, which, considered as 2-cells, are pieces of surfaces with given boundaries.

For instance, the category of categories itself is naturally a (strict) 2-category: the horizontal arrows are the functors and the 2-cells are the natural transformations between functors. There are then two compositions of morphisms, the horizontal composition of 2-cells for two composable functors, f:T → T', f':T' → T”, and the vertical one for three functors f, g, h from T to T'. These two compositions, are associative and compatible, and there exist natural “units” U_T: Id → F(T,T), analogs of neutral elements in a category. Taking these properties for axioms, we get (so-called strict) 2-categories. However, a notion of a weak 2-category is preferable in many respects; it imposes the associativity of horizontal composition only to the extent of isomorphism.

The same happens for morphisms of stacks, and for fields of stacks over sites (cf. Giraud, 1971). Taking for objects the geometrical toposes of the motion spaces, and for morphisms the functors compatible with the geometries, we obtain a notion of geometrical 2-category, applicable to networks of networks in the brain. Such a geometry can serve to adapt the dynamic of one action space to another one when coupling actions.

An example is a geometry corresponding to the role of the thalamus in the sensory motor process. The thalamus unfolds the spaces for forms, colors etc. The thalamo-cortical connections select and distribute the preferred characteristics of neurons in cortical areas cf. the “black-board theory” of Mumford (1991). Moreover, the various nuclei of the thalamus are linked via the cortex, generating specific oscillations (Llinas, 2002), and all send copies of activities to the motor system, giving a sort of sensori-motor web (Sherman and Guillery, 2002).

Example: let us consider locomotion and prehension together: to keep an object when walking. The brains networks controlling the two tasks is known, and there exist experimental results about their coupling (Ivanenko et al., 2005). It appears that the five main independent components of muscular synergies in locomotion are preserved in the coupling but with different weights, and one component is added by the task. Then the supplementary task influences locomotion changing weights of the synergies without changing its fundamental aspects, and transforms its own dynamic. We propose that the prehension system influences the locomotion system through reciprocal connections with the thalamic nuclei. In terms of toposic description, this corresponds to the schema of oriented product of the two toposes over a topos of the Thalamus (Giraud, 1972). This can be modelized by 2-stacks over 2-topos.

#### The level 3

An example of level 3 is given by the action of the cerebellum on the whole brain activities. The cerebellum is now known to have a cognitive role, in addition to its role in movements preparation and their control in real time, plus learning and so on. For instance, in the article of Heck et al. (2023), it is suggested that the wide projections of the cerebellum to the thalamus, has for function the stabilization of the synchronization of oscillations at large distance between cortical activities, that are established in great part by interactions with the thalamus.

Another example is given by Thalamo-basal-ganglia- cortical networks (cf. Squire, 2013). Several large loops in the brain have been proposed: (a) a dorsal BG regions with a specifical oculomotor loop; (b) a more cognitive loop, e.g., for language learning, homologous (by convergence) to the pallium-striatum loop for learning to sing in birds; (c) an emotional loop, involving rather ventral BG regions. And so on. However, there are many reciprocal connections between the loops (Hintzen et al., 2018).

This invites us to consider the mathematical structure that brings together 2-categories, within a 3-category: between two objects (here, complete loops open to action) the arrows are 2-functors (respecting the second-order structure) which themselves constitute a 2-category, with horizontal arrows and vertical 2-cells; moreover, there are 3-cells to compose these arrows and cells.

The 3-cells, homotopy classes of homotopies of homotopies, are the new elements; in our neural interpretation, they involve large loops through the internal connections to the central nuclei and cortex. If this conjectural model holds, it entails remarkable compatibility relationships, which neuromodulators must maintain.

It seems that 3 already permits a lot of combinations, and 4 is not necessary. This unique geometry organizes combinations of brain's networks. This system involves sensory-motor networks in the cortex, related by thalamic connections then linked and covered by connections between subcortical structures, colliculus, cerebellum, basal ganglia, cortical networks, all these systems being alimented and adapted by convenient varying diffusions of neuro-modulators.

Then our suggestion is compatible both with *modularity* of the brain (Changeux et al. op.cit.) and *re-entrance* (Edelman et al., 2011). Action spaces involve specific networks of areas (related to distinct action/perception spaces) distributed over the brain (in general between three and ten areas), but higher geometries involve several of these specialized networks. This level may bring together, without mixing them, action, cognition, emotion, memory, etc. like a 3-symphony.

Remark: The different levels correspond to different kinds of geometries, simply because they do not relate to the same levels of structures. Let us take an analogy: At level 1: People exchange ideas, at level 2: several people observe several groups of people talking to each other, and try to integrate (by exchanging information) these level 1 exchanges, for example to synthesize them, but perhaps also to help a group with the remarks of others. At level 3: the supervisors observe all this, to help the level 2 observers to do their job. Complexity evidently grows with the level.

## A geometrical theory of beauty

The core of our theory of beauty is that perception, judgment, and experience of beauty are possible when the brain can process the characteristics of the object with these high-level geometries. Then we propose that there are at least three levels in this dynamic processing, corresponding to the three level of geometries described above.

Our suggestion therefore is that beauty reflects the embedding of a subject of interest (ideal or existent) in a higher structure of spaces of the inner world of the brain. This relates beauty judgment to a coincidence between outside and inside world. Moreover, in general the local geometries come (directly or indirectly, by transposition) from motion spaces, then beauty is constantly linked with movements. In each case, beauty is the internal access to higher geometries, involving generalized parts of a whole, not of things but of relations between things or events and thoughts.

In this section we shall give a few examples which we believe support the theory proposed above. Most of the examples of experience or judgment of beauty are related with the ordinary geometry of ambient space. But every reference in the brain to the ambient space is dependent of a space of movement (cf above quotation of Poincaré). Thus, we suggest that the beauty of forms, visual, tactile, auditory, depends upon their relation with movements, mostly voluntary movements (cf. what was said by Saint François de Sales mentioned in the introduction), where preparation and adaptation are used.

Almost all examples of experience of beauty can be related to all the three levels of geometries and neural systems. Higher is the level, higher the feeling of beauty. Then for more pleasure we certainly try to access higher levels. Certain experiences go immediately to level 2 or 3. Other examples are purely of level 1, because they mainly rely on one particular geometry, through a group G. Example: an ellipse, it relies on affine transformations that preserve the ellipse, and other affine transformations that send this ellipse to another one. And a simple motion on the arc of this ellipse is juged beautiful when it obeys the 2/3 law. However, the ellipse alone is something too poor and abstract for generating a full feeling of beauty. An example that enriches the feeling is given by the consideration of the ellipses drawn by Piero della Francesca for representing the aureolas in his paintings, like *The Annunciation* (polyptique de Saint Antoine de Padoue). The cultural and pictural context can lead the experience at level 3.

The first level of beauty related to geometrical properties concerns “geometry” in the classical sense. It covers the characteristics of beauty given, as mentioned in the introduction, by Plato and Aristotle, who relied on symmetries or “proportions” between the elements of a whole.

### The beauty of a high jump

The perception of beauty when watching a high jump or even a picture of it, may come from the successful adaptation between the body motions and the obstacle. The topos geometries can internalize the dynamic properties of the object. For sport, several individual action spaces can describe different sports. They correspond to specific strongly adaptated learnings. Then they probably are of *level 1*. But they attain higher levels when comparing with other performances, or when embedding in special context.

At the second *level*, we have the unfolded spaces of movements all involved together, linked by “functors”; example: the harmony of all the segments of a human body tending toward the same goal of embracing another.

### The golden ratio

It is the unique ratio between the lengths of perpendicular segments, which is stable under the operation of subtraction of a largest inscribed square. Its recurrent appearance in architecture is known, but this number also appears frequently in nature, for instance in phyllotaxis, and convincing explanation were found recently by Douady and Couder (1996). Clearly this ratio refers to Euclidian geometry, but proportions theory overlaps Affine geometry, and more deeply, the infinite process of dividing the rectangle describe a spiral, belonging to Conformal geometry. This relation between three groups and asymptotics is at the *level 2*.

### A beautiful body

The body is always represented in various postures which, as Nicolai Bernstein wrote, is “preparation to act.” What is therefore immediately perceived is the graph of relations between body parts as for example in bodily expression of emotion (de Gelder, 2016) or the repertoire of coordination of head, arm and legs in the basic postural synergies (described by Fukuda, 1961, for instance). Underlied social interactions put it at least at level 2, probably 3.

### A beautiful decision

Elegantly (economically) solving difficulties, respecting symmetries or acting on them (cf. Gordian knot of Alexander). In this case, the reference space itself has to be invented for containing the action, and what is changed by the event must possess some evidence. The higher structure here is at least of *level 2*, because the decision, to be beautiful, must realize a transformation of stories, some of them being at level 1.

### The Cistercian square

Form of building propagated, elected by St Bernard in the architecture of convents. It manifests agreement between the thoughts, the actions and the form, then it also has something of level 2. Beauty comes from the many relations between these entities.

### A beautiful face

What makes a face beautiful or attractive is a very old and debated subject. See the summary or researches “Contribution of the visual system” in the introduction. Surely it involves a delicate balance between symmetry and harmony of many proportions, which evokes a complex geometry of level 1, but it is not sufficient in general. For instance, the expression in the eyes, the mouth, the chin and other parts, plays an important role for augmenting the feeling of beauty. This combines the judgment of beauty with other impressions and emotional feelings, like fear or joy. An example is the activation of the fusiform gyrus when we look at a face without exchanging gaze and the additional involvement of the amygdala, which activates the emotional system, as soon as we look at the other looking to us. This induces a great variability in the experience, and eventually the judgment, of beauty. Remark that subtile artificial technologies can generate all the characteristics of a beautiful face, this gives apparently a geometry of level 1 (where for instance harmony requires generally a lack of excessive symmetries). But this cannot be interpreted as a complete geometry for beauty, because what matters is always the inner geometry, not the external reality. And this geometry for the faces, involves in general not only forms and moves but also emotions and social values, relying on other loops in the brain. Therefore, in general the geometry underlying the experience of beauty of a face is of level 3.

### Beautiful music

The harmony in a concerto (or even a symphony) of all the instruments animated by all the musicians, the acoustics of the hall and its visual splendor. A beautifull opera evokes the correspondence between a spectacle, songs, movements, scenery and a story, romance, drama, feelings; this implies more than one topos. Similarly, a *fine film*, a *fine novel*, respectively, different from a good film and a good novel, which make for a good time; une *belle vie*, the song says *ah la belle vie!* Surely at level 3. This does'nt forbid a basic geometry at level 1, taking various forms depending on cultures or fashions, whose principles appear in the various rules in musical composition.

### The paintings of Chardin

As we have chosen to present in more details the geometry in the sense of topos of the reaching and grasping space, we suggest to have a look at the famous paintings of Jean Siméon Chardin, around the middle of XVIII th century. Many of them represent persons, children, young ladies or women and painters, taking objects in their hands, or touching them with their eyes (Maurice Merleau-Ponty said “vision is palpation by gaze”). For instance, “L'enfant au toton” painted before 1738, a very young noble child looking at a spinning top, hesitating to keep it in the right hand. The form and movement of the goal is familiar, such that we understand its instant properties, and also memorize personal experience with them; however, we are immediately also attracted by the hand, delicately semi-open, rested on the table, well-prepared for a future movement of grasping, helped for that by the angle of the arm in space. But our attention is also attracted by the body, although vertical, we feel its inclination to the table and the spinning top, and we are also interested by the semi-closed eyes. Note that quickly, we feel an ambiguity: the child has just put the spinnin top in motion, and is withdrawn by contemplation, which augments the scene in the past.

All these parts are in dynamic relations, aside from the main scene, object plus hand ready to start. The scene is a whole, but we feel peripheral elements as essential, which is an occurrence of the morphisms between the space-time of the main scene and the other ones, like body, head, eyes. Perhaps very important is the atmosphere in the room, the contrast with the immobile books and the interesting forms in the desk drawer, the harmony with the colors of the back wall, the four vertical red lines on it, and the living attitude of the child.

This experience of beauty is evidently at *level 3, even if the movement of level 1 remains central*. Many other paintings of Chardin can be analyzed in the same way, for instance, “Le dessinateur” taking and shaprpening its pencil, or “Le chateaux de cartes,” or “La jeune fille au volant.” All being examples of gestures beauty.

## Discussion

In the present text we provide previous evidence that the brain uses for perceiving and acting, a variety of geometries made for the production, adaptation, and control of voluntary goal oriented actions, requiring movements. We also suggest that the brain provides prolongations and transpositions of these geometries in thinking, remembering and other domains, and use all of them for feeling and experiencing or judging beauty. We have proposed a mathematical model inspired from Alexander Grothendieck, but adapted by one of us (D.B), of generalized spaces for these geometries, based on Topos, Stacks and n-categories. And we have suggested that the sensation of Beauty coincides with the feeling of participation of something in the world to these inner geometrical spaces. Given that we cannot at his stage provide empirical demonstration of the validity of our theory. Clear limitations of this theory can be mentioned. We shall list a few of them.

First, we do not have yet described precisely the inner geometries of all the motion spaces, or observed them experimentally. A computational model should bee developped in the direction of the remarkable work of Michaels et al. (2020) for reaching.

Secondly, we would have to consider the emotions produced by these higher level geometries, more or less directly related to movements, sensations and perceptions in space.

Thirdly, we have, in the first part of this article, mentioned the existence of several action spaces (body, peripersonal, near locomotor and far, environnemental) and proposed that different geometries are involved in the brain for the treatment of perception and action in these different spaces. Moreover, Berthoz (2020) has proposed that in painting one can very often observe the use by painters of at least three canonical spaces. The link between this modularity and the three levels described above still has to be established.

Our theory is compatible with some previous philosophical and neuroscientific theories. We agree with Plotin. For him, the judgement of Beauty relies on the elevation to Reason. This is similar to our suggestion: elevation to a higher order of dynamical structures. We already mentioned that our theory extends the oldest theories of Plato (insitsing upon *proportions* and *colors*), of Aristotle, Stoicists and Epicurians (privileging *order and measure*, and measurements comparison), and of Diderot on perception of *natural ratios*. We also integrate parts of the Hogarth criteria in relation to motion, with respect to his “Six principles” which are, according to him, necessary or helpful conditions of beauty: *Fitness, Variety, Regularity, Simplicity, Intricacy, Quantity*. Only Variety is clearly compatible with our theory because it implies the embedding in higher dimensional spaces, but the other ones are also directly related to one of the main property of the Topos type geometries which is *adaptation*.

We agree with the discoveries of Brielmann et al. (2021). Our suggestion that to experience beauty, we must access a higher order of intelligibility, explains her concepts of “*universality*,” “*exceeding*,” “*harmony in variety*,” and “*meaningfulness*.” The two last aspects “*pleasure*,” “*persistence*,” belong to the emotional world, and are more behavioral, in the sense of James (1950). There is in all these concepts a difficulty which is the dependence of the judgment on social and other contextual factors, like it is when we want to qualify a gesture as friendly or violent (Chartier et al., 2017).

One of the limits of the present work concerns the difference between the universal and the particular individual experiences of beauty. In our theory we have insisted upon the universality of the geometrical and dynamical features which underly the essence of objects, forms, scenes, a.s.o. that can access to the general category named beauty. However, it is of common knowledge that there is a great diversity in the way humans perceive, experience and sense beauty. Indeed, at the outset of this article we recalled that shared (universal) taste contributes only 19% to beauty-rating variance, while personal taste contributes 3 times as much, and we concluded that beauty is more personal than universal. It is known that in different cultures, different things are considered as beautiful. In contrast to bodily action in space, some judgments are deeply rooted in the historical community (and even action in space may depend on some historicity, but it surely has a more “humanly universal” nature). For example, it is hard to identify Chinese appreciation of beauty and European, even less in music: history makes them very different. Example: major and minor ranges based on octave vs. pentatonic sequences.

Then, our theory can appear as an excessive “universalization” of aesthetic judgments. The higher geometries that are envisaged as the abstract substrates of movement generation as well as of aesthetic judgments are presumably an invariant property of all brains. This is compatible with the universals of both motor planning and aesthetics. But how can we reconcile these invariant properties with the variable, individual features of both movement generation and sense of beauty?

But we insist here on the fact that the various generalized geometries used by the brain contain themselves the power of diversity. Whatever being the level of generalized geometry, 1, 2 or 3, a multiplicity of specific solutions can be installed and used. At level 1 already the coordination of movements and the laws of movement, because they involve dynamic relationships between kinematic variables, may allow a variety of concrete generating different “styles,” depending on contexts and individual subjects. However, surely the variability grows with the level of geometry.

In other words, it seems to us that there is no *a priori* opposition between our general theory and the diversity of the concrete implementations in our brain of the experience and judgment of beauty. However, we have not been able in this paper to account precisely for this important issue which is also present in the opposition, for instance, in paleontology between genotype and phenotype, and in the distinction between simplexity and vicariance (Berthoz, 2012, 2016). Further work based upon empirical attempts to confirm our theory in various circumstances may provide a better understanding of this fascinating challenge.

A recent study (Brielmann et al., 2023) has modeled the existence of particular individual judgments of beauty over time by using a form of predictive optimal coding based on fluency and efficient learning. The method uses an artificial deep neural network (implementing a neural crossbreed morphing algorithm) for measuring liking judgments of images. In this model, there is no explicit consideration of the brain functioning or of geometry, however it could be fruitful for us to compare with our theory, as it was useful to combine optimization and statistics with the geometrical approach for better understanding hand movements (cf. Flash et al., 2016; Zago et al., 2018).

Our approach could be judged as tautological in the sense that we suggest that the experience of beauty occurs when appears a common activation of some neuronal assemblies in which particular geometries are installed. But we have not proposed how the conscious knowledge of experience and judgment of beauty arise. This is similar to the general question of conscious experience as discussed by many contemporary authors that we cannot review here. A potential candidate is the activation of neuronal assemblies specific of the involment of *the self* in presence of some thing, or event. Because experience of beauty is known to involve a unique link with the circuits of self-reference and sel-relevance. See the recent paper study by Salgues et al. (2021). Their results suggest that a basic mechanism, appraisal of self-relevance, could ground aesthetic judgments. Then we conjecture that self-relevance is active when geometries are installed in specific networks, and continues to be active when feeling geometry and beauty, that concern our own integration in the world.

## Data Availability

The original contributions presented in the study are included in the article/Supplementary material, further inquiries can be directed to the corresponding author.
