# Supplementary material for: Brain's geometries for movements and beauty judgments. A contribution of topos geometries
Source: Front Psychol. 2025 Sep 4;16:1583185. doi: 10.3389/fpsyg.2025.1583185 (PMC12444762; doi:10.3389/fpsyg.2025.1583185)
Supplement: Supplementary file 1 [file Data_Sheet_1.docx]

**Supplementary document**

**Appendix 1. Basic concepts of Topos geometry**

A (mathematical) **group** is defined as a non-empty set endowed with an internal binary operation, which is associative, possibly commutative, and has a neutral element (the identity) and inverse elements. In particular, the group of displacements of rigid bodies, which includes translations, rotations and twists in Euclidean space, preserves (metric) distances and angles. Reflections in a mirror are not displacements, but preserve lengths and angles; the complete group of Euclidean geometry is made up of displacements and their composition with reflections. Other groups define other classical geometries, like affine or equi-afffine. The affine geometry forgets the notion of size and of angle, but retains the notion of paprallelism of straight lines. Its group can be concretely represented, acting in a vector space, by the invertible linear transformations composed with translations. The equi-affine geometry corresponds to the sub-group of transformations preserving the volumes (i.e. the aresas in 2D). A different example is the group of permutations of a set of symbols.

A **category** is a generalization of a group; it is made up of a collection of objects and sets of arrows between pairs of objects (named morphisms), which can be composed associatively, and have a neutral element for each object. A groupoid is a category where each arrow is invertible. In particular, a groupoid that has a single object is a group. Examples of groupoids that are not groups are given by non-holonomic displacements from one configuration to another, such as the movements of an arm or a leg; here the objects are the configurations and the arrows are the body transformations (note that they are invertible, but cannot be composed indefinitely). An example that illustrates well the notion of category in general, different from groups or groupoids, is the set of all rectangular matrices (with numerical coeffficients), where objects are the integer numbers and the morphisms between two such integers n and m, are the matrices with n colmuns and m lines. This captures the elements of linear algebra in finite dimension. The diagonal matrix of size n with 1 along the diagonal is the neutral element at n.

**Functors:** categories can be transformed into categories using specific structure-preserving transformations, called “functors”, and different functors can be compared using “natural transformations”. Category theory can appear as a general abstract diagrammatic language and as a unified, fundamental framework for all areas of mathematics. A nice reference is (MacLane S, 2010). The *opposite* C^op of a category C is simply obtained by reversiong the arros of C. A functor from this category to another category is a *contra-variant* functor on C.

**Topos**: in simple “logical” terms, a **topos** is a category where it is possible to make all the known constructions in set theory: products of objects, objects of morphisms between objects, and where there is a good (natural) notion objects of sub-objects of each object, satisfying the same relatins than do sets in set theory. Cf. (Prouté, 2010), (Bell, 1988), (Caramello, 2017). Then each topos provides a generalization of set theory, and can be used to build a contextual logic, like a Boolean theory, but without the usual principle of excluded middle. However, most interesting toposes have also a geometric interpretation (very important in practice); they are the Grothendieck toposes, attached to set theory: equality of morphisms can be tested on a set of arrows, and there is a functor from the topos to the category of sets, that respects the logical operations. For such a topos T, there exist a category C such that any object of T is a contra-variant functor from C to Set, satisfying a collection of constraints, given by a topology on C in the sense of Grothendieck. We describe now what it means precisely.

**Grothendieck Topologies and Sheaves. Définitions**

Although apparently technical, this notion merits the attention of neuroscientifics, because it concerns the natural organization of connexions in a network, which can support a coherent local set theory, that is a basis for reasoning.

A **refinement R**, or sieve (cribble in French) of an object a in a category C, is a colection of arrows f:b→a of C, such that for any arrow g:c→b, and f in R, the arrow fog:c→a belongs to R. For any morphism h:b→a, we define h*R as the (**pullback**) refinement of b, given by the arrows g:c→b, such that fog:c→a belongs to R.

A Grothendieck topology J on C, consists in the choice of a family of refinements J(a) of a, for all the objects of C, satisfying the following three axioms: (i) for f:b→a belonging to an R in J(a), the pullback f*R belongs to J(b); (ii) id_a belongs to one R in J(a) (this means that the refinement by all the arrows C|a abouting to a belongs to J(a)); (iii) for any a in C and R in J(a), another refinement R’ of a also belongs to J(a) if and only if, for every f:b→a of R, f*(R’) belongs to J(b). A category C equipped with a topology J is named a **site**. The elements of J(a) are named the coverings of the object a.

A contra-variant functor F from C to the category Set is named a **presheaf**. If R is a refinement of a in C, we consider the induced presheaf F|R, and define lim(F|R) as the subset of the product set ∏F|R of the F(b), for all f:b→a in R (the produt being indexed by the morphsims f), whose elements {x(b)}_(f in R), satisfy F(g)(x_b)=x_c, for every g:c→b. In other terms lim(F|R) is the set of coherent sections of F over R. (In the applications to neural networks, F represents possible dynamics in time of each area, and the limits correspond to the compatible dynamics over the areas.)

If J is a topology on C, a presheaf F on C is said to be a **sheaf,** when for every a in C and R in J(a), the natural map from F(a) to the set ∏F|R induced by the arrows f:b→a in R is an isomorphism. Therefore F(a) is a set of coherent sections over R. A **Grothendieck topos** is any category isomorphic to the category of sheaves over a site.

The leading example, justifying the axioms, is given by the category C of opens sets in a topological space X, with inclusions as morphisms, where the refinements are the usual coverings of open sets by open subsets. Another kind of example is given by all the the classifying topos of a group G, seen as a site with the trivial topology; in fact, a sheaf is nothing else than a set equipped with an action of G (to the right).

In practice, only few sheaves are considered on the site C of the topos T, but it is more elegant to consider all possible functors on C, then forming the whole topos, for instance to make usual sets constructions (as sets of sets). For instance, in our applications, the arrows F(b)→F(a) of one object F of the topos T (over C equipped with a topology) represent neuronal connexions between brain areas b and a, then different sets of possible activities in the network are represented by different objects in the topos. Then in learning, or development, many different objects can appear, then at least a part of the whole topos will be necessarily explored. Also, by considering the topos, the fact of modifying connexions becomes accessible to the model.

**Toposes Geometries and Fields of Stacks. Definitions.**

A **geometrical space** according to Klein (Erlangen program), is a set equipped with a group of transformations that can send every point to any other given point. A **stack** S is a family of such geometric spaces and transformations between them, indexed coherently by a category C (its site) thare is compatible with the acting groups (and compatible with J). We will also consider the generalized version, where local groups are replaced by arbitrary categories, for instance groupoids or hierarchical structures for classifications, described by trees. We write S→C, and name it a fibration of categories, cf. (Giraud, 1971). In the generalized case, even for groupoids, we can replace the actions on sets by contravariant functors between two categories.

We will also consider the collection of stacks with a given site and internal structures as a **geometrical topos**, because once a stack is given, it is advantageous to consider all the stacks having the same site, and the same geometry of the fibers, but varying their relations. This enlargement from an object to the whole family of stacks over the site generalizes the passage from a pre-sheaf over a category C to the topos of sheaves over C.

In fact, this notion of **stack** (or **champ** from mathematical French) is the more fundamental element of structure for us, to describe brain’s geometries, associated to movements or not. The category which is the basis of the stack is a **site**. The topos T_S of pre-sheaves on a stack itself S can be seen as a generalized topos of pre-sheaves on C with values in toposes over the fibers. The obects of T_S are themselves stacks over C. Cf. (Giraud, J. ,1972).

# **Appendix 2.** **The grasping (and reaching) topos**

Let us describe more precisely what the brain’s network of grasping is, and how we transform this network to form a topos, then a stack. This is made in four steps: 1) construction of a shadow-graph, embedded in the 3 dimensions of the brain ; 2) construction of a graph in space time, representing the dynamis, lifting the step1; 3) introduce some kinds of canonical surgeries for constructing a site category C’_0 (with a topology) such that the dynamic forms a sheaf X_0 on it; 4) find a simpler model C with trivial topology, and presheaf X, having the same topos T. In particular, this allows to represent the learnings by morphisms in T.

This is an example of what is developed in the first chapter of Topos and Stacks of DNNs, (Belfiore, Bennequin, 2022).

The neurophysiological basis of the following simplified discussion of grasping in monkeys, was established by (Michaels et al. 2020). In this article, the authors prove experimentally, that the recurrent network reproduces fairly well the activity of cells in AIP, F5, M1 during grasping different forms of objects. They describe in particular the steps 1 and 2 below (in other words

1. ***Shadow-graph*** cf. Fig 1. A curved path goes directly from occipital visual areas V to parietal AIP, another longer path goes from V to AIP through the temporal cortex; the first is schematically for “where”, the second for “what”. That gives features vectors to AIP. Reciproval connections are represented by two edges between AIP and frontotemporal F5, premotor for hand and arm, and by two edges between F5 and primary motor area M1. Finally a longer path starts from M1 and goes to the chord and musles;
2. ***Space-time oriented graph*** cf Fig. 2. A grid of points in the vertical plane with three horizontal lines in the plane and an arrow of columns from 1to K (K large), plus a set of triangles in perspective joining points on a horizontal line to the vertices in the plane. This last line is for the “hold signal” (important spontaneous activity driving the dynamics). The planar vertices are named a_k, a’_k, a”_k (or a_i,k, i=0,1,2. Horizontal arrows go from one vetex to the next one to the right, they are the memory edges. Vertical arrows are bottom to top, from Inputs (features of objects) to a, then a to a’, a’ to a”, and a” to Output (motor signal). Diagonal up to bottom right are feedback connections, known to be also very important in the model. Transversal arrows go from front points to the three lines of central vertices. The figure 3 shows the generic star of arrows at vertices: four incoming Hold, Mem, vertical, Diag of feedback, and three outgoing, Mem, feedback, feedforward. The category freely generated by the space-time oriented graph is denoted by C_0.
3. ***Surgery. Forks and Grothendieck topology.*** Divergent (or outgoing) oriented edges are just reversed in C’_0 with respect to C_0. Convergent edges, from say x, y, …, to x’,…, pose a problem, because they underly transmission of activites of the form x’=F(x,y,z,…),… that cannot be represented by a pre-sheaf over C_0 (or the opposite category C_0^op). Therefore, we introduced two new vertices A*, A, new objects of C’_0, that are not in C_0. We maintain, without inversion the arrows from x, y, …, but now going to A*, then add an arrow from A* to A and an arrow from x’ to A (which plays the role of inversion of orientation). Cf. Fig 4. The non-trivial sieve of the topology is the set of arrows from x, y, …, to A*. This forces that in a sheaf X over C’_0, the value X(A*) is the product set of the sets X(x), X(y), … The old dynamic is X(x’→A). And we impose that X(A*→A) is the Identity, or at least an isomorphism.
4. ***Transformation in presheaves.*** It appears (Belfiore and Bennequin, 2022) that the site C’_0 defines the same topos as a simpler category C without non-trivial sieve. It consists to impose A*=A and to go directly from x’ to A. Fig. 3. Another model with the same number of points is given by a finite topological space. Fig. 5. (A is the place for free descions, or inner contributions.)

To get a stack, we include geometries in fibers over each object of C, at a this describes the transformations of the properties of the goal (texture, shape, weigth, …), at a’ the configurations of the hand (a non-holonomic geometry) and at a” the kinematics in the external world. At the new points A* (or A), this is a part of the product of the three, representing a form of compatible triples.
